# Fermentation of Blackberry with *L. plantarum* JBMI F5 Enhance the Protection Effect on UVB-Mediated Photoaging in Human Foreskin Fibroblast and Hairless Mice through Regulation of MAPK/NF-κB Signaling

**DOI:** 10.3390/nu11102429

**Published:** 2019-10-11

**Authors:** Ha-Rim Kim, Da-Hye Jeong, Sol Kim, Sang-Wang Lee, Hong-Sig Sin, Kang-Yeol Yu, Seung-Il Jeong, Seon-Young Kim

**Affiliations:** 1 Jeonju AgroBio-Materials Institute, Wonjangdong-gil 111-27, Deokjin-gu, Jeonju-si, Jeollabuk-do 54810, Korea; poshrim@jami.re.kr (H.-R.K.); jdh1017@jami.re.kr (D.-H.J.); solkim@jami.re.kr (S.K.); kangyu@jami.re.kr (K.-Y.Y.); sijeong@jami.re.kr (S.-I.J.); 2 Chebigen Co. Ltd., Jeonju 54853, Koreashsdoo@hanmail.net (H.-S.S.)

**Keywords:** skin aging, photoaging, fermented blackberry, *Lactobacillus plantarum*, type I procollagen, MMPs

## Abstract

Chronic and extensive exposure of ultraviolet (UV)-irradiation causes human skin sunburn, inflammation, or photoaging, which is associated with downregulated collagen synthesis. This study investigated the effects of fermented blackberry (*Rubus fruticosus* B., FBB) by *Lactobacillus plantarum* JBMI F5 (LP) on UVB-induced photoaging in human foreskin fibroblast (Hs68) as well as in SKH-1 hairless mice. FBB pretreatment inhibited UVB-mediated type-1 procollagen degradation, matrix metalloproteinase (MMP)-1 and MMP-2 protein expression, and suppressed nuclear factor-κB (NF-κB) activation as well as mitogen-activated protein kinase (MAPK) phosphorylation in Hs68. In addition, FBB administration diminished the wrinkle formation in dorsal skin and epidermal thickening in UVB-irradiated hairless mice. Moreover, UVB-induced Type-1 procollagen reduction and antioxidant enzyme inactivation were reversed by FBB administration. These results suggest that FBB may have antiphotoaging effects on UVB-induced wrinkle formation by maintaining the extracellular matrix density in the dermis, which occurs via regulation of reactive oxygen species and related MAPK and NF-κB signaling. Therefore, FBB can be a potential candidate for protecting skin aging against UV irradiation.

## 1. Introduction

The skin is the outermost part of human body and constantly exposed to potentially harmful compounds and hazardous environmental factors including solar ultraviolet (UV) radiation. Chronic solar UV exposure has deleterious effects on human skin, including erythema, immune suppression, cancer, and photoaging [1]. UV irradiation changes the dermal collagen through the stimulation of collagen breakdown and inhibition of procollagen biosynthesis, causing loss of collagen content. This process is mediated by enhanced matrix metalloproteinases (MMPs) expression that are responsible for degradation of extracellular matrix (ECM) proteins, which form skin dermal connective tissue [2]. Type I collagen is the most abundant protein in skin connective tissue. Type I procollagen is secreted into the dermal extracellular space to form regularly arranged fibrillar structures [3]. Oxidative stress is one of the most important causes of aging process. UV-irradiated skin generates intracellular reactive oxygen species (ROS) and reduces antioxidant enzymes including catalase and superoxide dismutase (SOD), and glutathione peroxidase (GPx) causes DNA damages and alteration of cellular homeostasis [3,4]. ROS, which is produced by UV irradiation in skin, induces an inflammatory response that is the expression of cyclooxygenase-2 (COX-2) and inducible nitric oxide synthase (iNOS). These ROS then stimulate the activation of mitogen-activated protein kinases (MAPKs) such as extracellular signal-regulated kinase (ERK), c-Jun N-terminal kinase (JNK), and p38 kinase, which induces nuclear factor kappa B (NF-κB) activation [5]. Applications of probiotics or botanical products in skincare products have recently increased due to their antioxidant and anti-inflammatory properties. Probiotics are known to modulate the immune system of the gut and to protect against infectious and inflammatory diseases by regulating intestinal microflora [6]. The role(s) of probiotics bacteria in dairy fermentations is to improve the release of free amino acids or the synthesis of vitamins and the provision of special therapeutic properties [7]. Recent reports have shown preclinical or clinical results of protection against atopic dermatitis, dry skin, and UV-induced skin damage by probiotics or combination with the active ingredients of natural products [8,9,10]. Blackberries are anthocyanin-rich fruit belonging to the family Rosaceae and display potent antioxidant and anticancer properties [11]. Recent reports shown that blackberries and their active compounds, such as anthocyanins, inhibits UV-induced oxidative damage and inflammations in keratinocytes and SKH-1 hairless mouse skin through MAPK and NF-κB signaling [11,12,13]. To the best our knowledge, no studies have considered the use of *L. plantarum* JBMI F5 (LP) fermentation to improve the functional features of blackberries (BB). In this study, we investigated the protective effect of fermented BB (FBB) on UVB-induced photodamage in human foreskin fibroblast (Hs68) and using the SKH-1 hairless mice. 

## 2. Materials and Methods

### 2.1. Chemicals and Antibodies

Phosphate-buffered saline (PBS), Dulbecco’s modified Eagle’s medium (DMEM), fetal bovine serum (FBS), and antibiotics (amphotericin B, penicillin, and streptomycin) were purchased from Invitrogen (Carlsbad, CA, USA). Folin reagent, (+)-catechin, sodium carbonate, gallic acid, metaphosphoric acid, and other chemicals were obtained from Sigma (St Louis, MO, USA). The pro-collagen type I (cat#. ab210966) and MMP-1 (cat#. ab100603) enzyme-linked immunosorbent assay (ELISA) kit was obtained from Abcam (Cambridge, UK). The primary and secondary antibodies used in Western blot analyses were purchased from Cell Signaling Technology Inc. (Beverly, MA, USA). All other chemicals were of analytical grade or complied with the standards required for cell culture experiments.

### 2.2. Preparation of FBB 

*L. plantarum* JBMI F5 (KACC91638P) was grown in MRS (de Man, Rogosa, and Sharpe) broth (Difco, USA) at 37 °C for 12–16 h. The cultivation media were suspended in sterile distilled water to a final optical density of 0.8 at a wavelength of 600 nm. After weighing the BB, water was added to make the blackberry content 66%. The raw BB materials thus prepared were mixed using a crusher. The crushed BB was filtered using a mesh. The prepared BB solution was adjusted to pH 6.0 for fermentation, sterilization, and then inoculated with 5% overnight cultured LP. The fermentation process was carried out for 7 h while stirring at 230 rpm, 37 °C. After the fermentation, the fermentation broth was recovered and added with dextrin, followed by lyophilization. The freeze-dried powder was stored at −20 °C.

### 2.3. Biochemical Analysis 

Total polyphenol and flavonoids contents (TPC and TFC) were analyzed using Folin Ciocalteu reagent and 10% AlCl_3_ [14]. Each content was expressed as quercetin or gallic acid equivalents (mg/g). Vitamin C (l-ascorbic acid) content (VC) was measured using the vitamin C assay kit (Megazyme, Bray, Co., Wicklow, Ireland) according to manufacturer’s instructions. DPPH (2,2-diphenyl-1-picrylhydrazyl) and ABTS (2,2′-azino-bis-3-ethylbezthiazoline-6-sulphonic acid) radical scavenging activity was determined by spectrophotometric method [14].

### 2.4. Cell Culture 

Human fibroblasts (Hs68) were obtained from the American Type Culture Collection (ATCC, Manassas, VA, USA) and cultured in Dulbecco’s modified eagle’s medium (DMEM) containing 10% fetal bovine serum (FBS), penicillin-streptomycin sulfate (100 units/mL and 100 μg/mL) at 37 °C with 5% CO_2_ atmosphere. Cells were maintained at culture densities below 1 × 10^5^ cells/mL.

### 2.5. Cell Viability Assay

Hs68 cells were plated 96-well culture plates and incubated with *Lactobacillus plantarum* JBMI F5 (LP), blackberry (BB), BB inoculated with LP (BBL), and fermented BB (FBB). After 24 h incubation, the cells were washed once with PBS and then exposed to UVB irradiation (100 mJ/cm^2^) using UVP Crosslinker (Analytik Jena AG, Jena, Germany). The distance between the UV light and the sample was 15 cm and the exposure time was 25 s/100 mJ/cm^2^. After UVB irradiation, cells were incubated in fresh culture media in the presence or absence of LP, BB, BBL, and FBB and further incubated for 24 h. After 24 h, cell viability was estimated using 3-(4,5-Dimethylthiazol-2-yl)-2,5-diphenyl tetrazolium bromide (MTT) assay. A 10 μL MTT solution (5 mg/mL) was added to each well, and the cells were further incubated for an additional 4 h. The supernatant was removed and the formazan was resolved with 100 μL of dimethyl sulfoxide (DMSO). The optical density was measured by microplate reader (Multiskan Go, Thermo Scientific, Waltham, MA, USA) at 570 nm. The values of control were considered 100% viable.

### 2.6. Animals

Six-week-old female hairless mice (SKH-1) were obtained from Samtaco (Osan, Korea). Mice were randomly divided into seven groups of three mice per cage and housed under standard conditions (22 ± 2 °C, 55 ± 5% humidity, and 12 h light/dark cycles). Mice were allowed to adapt to the conditions for one week before initiation of experiments and were free to consume water and food. The experimental protocol for this study were reviewed and approved by the Animal Care Committee of Jeonju AgroBio-Materials Institute, and strictly followed the committee guidelines (IACUC JAMI2018003).

### 2.7. UVB Induced Skin Damage Model in Hairless Mice

The mice were assigned to seven groups (*n* = 6), which are including the normal control (C), UVB only irradiation control (UVB), positive control (0.01% retinoic acid, RA), LP (1 × 10^10^ CFU), BB (158 mg/kg body weight), BBL (1 × 10^10^ CFU of LP and 158 mg/kg of BB), and FBB (1 × 10^10^ CFU of LP and 158 mg/kg of BB). Each sample was orally administered daily, 1 h prior to UVB irradiation. The bodyweight of mice was measured every week. The UV irradiation performed using UVP Crosslinkers (Analytik Jena AG, Jena, Germany) with a peak emission at 302 nm. UVB irradiation was applied to the back of the mice five times per week. The UVB was treated with 30 mJ/cm^2^ (1 minimal erythematous dose, MED) in the first week, and the dose was increased weekly by 1 MED until reaching 4 MED (120 mJ/cm^2^). After the experiments, the mice were sacrificed, and the dorsal skin was excised for histopathological studies. The blood serum was obtained by centrifugation at 3,000 rpm 15 min and then 12,000 rpm 10 min. Aliquots of serum samples were stored at −20 °C until analyzed.

### 2.8. Wrinkle Measurement

To determine the severity of wrinkles, each hairless mouse was anesthetized and the UV-exposed dorsal skin (wrinkle formation area) was photographed using a microscope camera. SILFLO impression material (Silflo, J&S Davis Ltd., UK) was used for to make dorsal skin replicas of the hairless mice. 

### 2.9. Measurement of Type I Procollagen and MMP-1

Hs68 cells (5 × 10^5^ cells/well) were seeded in six-well plates. After 18 h, the cells were treated with FBB at concentration of 1 × 10^7^ CFU/mL for 1 h at 37 °C in a 5% CO_2_ atmosphere. After treatment, exposed to UVB irradiation (100 mJ/cm^2^) using UVP Crosslinker, and the incubation was continued for a further 24 h. The culture medium was then harvested and the chemokine production levels in the supernatants were measured using ELISA kits according to the manufacturer’s instructions. The inhibitory effect of FBB was determined based on the absorbance at 450 nm, measured using an ELISA reader (Multiskan Go, Thermo Scientific, Waltham, MA, USA). Mouse MMP-1 (CUSABIO, Wuhan, China) and procollagen type I (Abcam, Cambridge, UK) were used to assess in mouse serum.

### 2.10. Determination of the Antioxidant Activity

Superoxide dismutase (SOD) activities were measured using a SOD assay kit (Sigma, MO, USA), according to manufacturer’s instructions. Briefly, 20 μL of serum and tissue homogenate was mixed with 200 μL of WST-1 (2-(4-iodophenyl)-3-(4-nitrophenyl)-5-(2, 4-disulfo-phenyl)-2H-tetrazolium, monosodium salt) working solution in a 96-well plate. Then, 20 μL of enzyme working solution was added to the mixture, and incubated for 20 min at 37 °C. The plate was measured at 450 nm using a UV spectrophotometer (Thermo Scientific, Germany). Glutathione levels were determined using a GSH assay kit (Cayman, MI, USA). Serum and tissue homogenate added triethanolamine solution and Assay Cocktail [a mixture of MES buffer (11.25 mL), reconstituted cofactor mixture (0.45 mL), reconstituted enzyme mixture (2.1 mL), water (2.3 mL), and DTNB (0.45 mL)], total GSH in the deproteinated sample was measured using a UV spectrophotometer at 412 nm.

### 2.11. Immunoblotting

Protein extraction and immunoblotting of cells and tissues were performed as previously [15]. Proteins (20 μg per lane) were resolved on a 10% or 12% SDS-PAGE gel and transferred to polyvinylidene difluoride (PVDF, GE Healthcare, Little Chalfont, Buckinghamshire, UK) membranes. Blots were incubated with primary antibodies (1:2500 dilutions of each antibody) overnight at 4 °C. The blots were rinsed four times with blocking buffer and incubated with horseradish peroxidase-conjugated secondary antibodies (1:5000 dilutions of each antibody) for 1 h at room temperature. The binding of the antibodies was visualized using an enhanced chemiluminescence (ECL) system (Bio-Rad, Munich, Germany). Protein concentrations were determined using a Bio-Rad protein assay kit and known concentrations of bovine serum albumin (BSA) were used as the standard. All immunoreactive signals were analyzed by densitometric scanning (Amersham imager 600, GE Healthcare, Buckinghamshire, UK).

### 2.12. Histological Analysis

Mouse skin tissues were fixed in 4% paraformaldehyde for 24 h, and embedded in paraffin. Serial sections (4 μm) were mounted onto silane-coated slides and stained with Hematoxylin and eosin (H&E) for general histopathology or Masson’s trichrome to detect collagen, respectively. For MMP-1 immunostaining, the Envision^TM^ staining kit (DAKO, was used according to the manufacturer’s instructions. Briefly, slides were deparaffinized and rehydrated. Peroxidases and avidines (DAKO, Carpinteria, CA) were blocked at 10 and 5 min respectively. Samples were washed in PBS, incubated with the primary antibody (MMP-1, Abcam, Cambridge, MA, USA), and with the HRP rabbit Envision^TM^ System (DAKO) for 1 h at room temperature. Antigen–antibody complexes were revealed by DAB (DAKO) and counterstained with Mayer’s hematoxylin. All stained skin specimens were observed and photographed using an optical microscope (Olympus, Tokyo, Japan). To quantify epidermal hyperproliferation following UV exposure, the vertical thickness of the epidermis was measured at five randomly selected locations per slide with 200× magnification (H&E staining). The data were evaluated through the image analysis program Image J 1.52 (Wayne Rasband, National Institutes of Health, Bethesda, MD, USA).

### 2.13. Statistical Analysis

Data represent means ± standard deviation (SD) of at least three separate experiments. Student’s t-test was performed to compare the parameters between two groups, while the analysis of variance (ANOVA) test followed by the Tukey post hoc test was performed to compare the parameters among three groups. Data with *p* < 0.05 were considered statistically significant.

## 3. Results

### 3.1. Biochemical Changes by Fermentation

The biochemical content and antioxidant effects were presented in Table 1. Measurement of each biochemical contents and antioxidant effects was evaluated in BB and fermented BB (FBB) with *L. plantarum* JBMI F5. As shown in Table 1, the TPC and TFC results was increased by fermentation. In case of vitamin C, the contents were remarkably increased over in FBB compared to BB. The Phenolic compounds usually have glyosidic bonds with a various glycoside, which bonds were hydrolyzed by microorganisms for the release of the total phenolics and flavonoids [16]. The phenolic compounds possess a beneficial effect on anti-inflammatory activity and related diseases. The antioxidant activity, i.e., DPPH and ABTS, was similarly increased in FBB compared to BB. These results suggest that BB fermented with LP may be more effective than BB for UVB-induced photoaging in skin due to its increased antioxidant effect and biochemical contents.

### 3.2. Protective Effects of FBB on UVB-Induced Damage in Hs68 Cells

**Figure 1:**
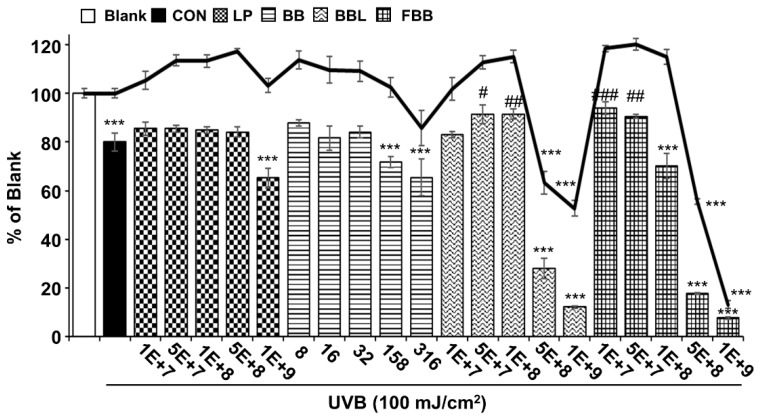
UVB-induced damage of normal human foreskin fibroblast (Hs68) was decreased by pretreatment with fermented blackberry (FBB). Cells were treated with various doses of *L. plantarum* JBMI F5 (LP), BB inoculated with *L. plantarum* JBMI F5 blackberry (BBL), and FBB for 24 h. Cytotoxicity was determined with MTT assay. The cell survival activity for cells with pretreated with different concentrations of LP, BB, BBL, and FBB for 24 h and irradiated with UVB (100 mJ/cm^2^) in Hs68 cells. The viability was analyzed using MTT assay. *** *p* < 0.001 vs. Blank; ^#^ *p* < 0.05, ^##^ *p* < 0.01, ^###^ *p* < 0.001 vs. Control (CON, UVB irradiated group). Bar; Cell viability, Line; Cytotoxicity. Values are means ± SEM of three independent experiments.

The LP, BB, and BBL were tested as controls for FBB. The cytotoxic effects of samples on Hs68 cells were measured by MTT assay. Hs68 cells were treated for 24 h with various concentrations ranging from 1 × 10^7^ to 1 × 10^9^ CFU/mL for LP, BBL and FBB and from 8 to 316 μg/mL for BB, which are equivalent doses of BB in FBB, the cell viability was reduced up to 1 × 10^8^ CFU/mL (Figure 1). As shown in Figure 1, UVB (100 mJ/cm^2^) irradiation significantly decreased the viability of Hs68 cells compared with that of the non-irradiated Blank group. When UVB-exposed Hs68 cells were pretreated with FBB at various concentrations, cell viability was significantly increased at doses of 1 × 10^7^ and 5 × 10^7^ CFU/mL compared to the UVB irradiated group. Therefore, the most effective and non-cytotoxic concentration of FBB, 1 × 10^7^, was used in subsequent studies. 

### 3.3. Regulation of UVB-Induced Collagens (COLs) Degradation and MMPs Expression by FBB in Hs68 Cells

**Figure 2:**
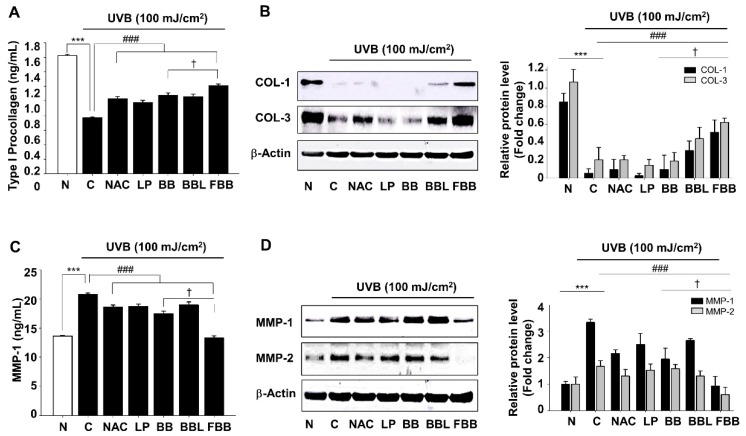
Effects of FBB on UVB-induced collagens (COL) degradation and matrix metalloproteinases (MMPs) production in Hs68 cells. Cells were stimulated with UVB (100 mJ/cm^2^) after incubated with the indicated doses of LP, BB, BBL, and FBB for 24 h in Hs68 cells. (**A**) Type I procollagen levels were measured in cell-free culture supernatants with ELISA kits. Cell lysates were analyzed by western blotting to determine the protein levels. (**B**) COL-1 and -3 protein levels. (**C**) MMP-1 levels in cell-free culture supernatants. (**D**) MMP-1 and -2 protein expression levels. The densitometry data represent are shown as relative density of protein bands normalized to β-actin level. *** *p* < 0.001 vs. normal (N, without UVB irradiation); ^###^ *p* < 0.001 vs. control (C). ^†^ *p* < 0.05 vs. BB. Values are means ± SEM of three independent experiments.

To assess the inhibitory effects of FBB on COLs degradation and MMPs production in UVB-exposed Hs68 cells, cells were exposed to UVB 1 h after FBB treatment. After 24 h, the secreted level of type 1 procollagen and MMP-1 was measured by ELISA. As described in Figure 2A, FBB substantially enhanced cellular levels of type 1 procollagen degradation by UVB. MMP-1 levels were enhanced by 1.5-fold due to UVB irradiation compared to non-irradiated cells (Blank). Treatment with FBB restored MMP-1 levels by about 97% (Figure 2C). To confirm whether FBB regulated COLs (COL-1 and COL-3) and MMPs (MMP-1 and MMP-2) expression in UVB-induced Hs68 cells, we evaluated the protein expression by immunoblotting. Figure 2B,D shows that the COLs were remarkably decreased and the MMPs significantly increased after UVB irradiation, FBB restored COLs degradation and MMPs overproduction. FBB showed more significant effect compared to non-fermented BB on UVB-induced type I procollagen degradation and MMP-1 levels increase. Interestingly, FBB exhibited more effective results than that of positive control, *N*-acetyl-l-cysteine (NAC), on COLs degradation as well as MMPs expression (Figure 2B,D). 

### 3.4. FBB Suppresses the MAPK and NF-κB Signaling in UVB-Exposed Hs68 Fibroblasts

**Figure 3:**
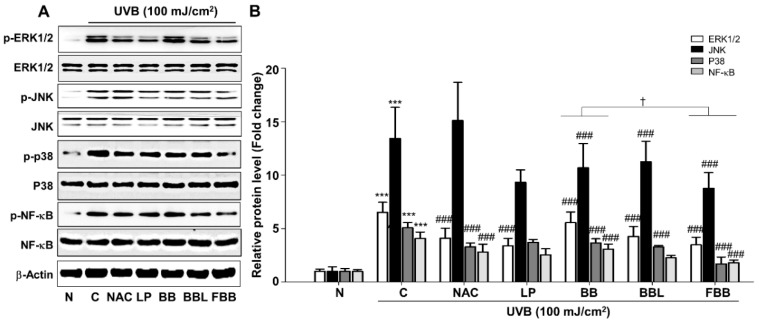
Effects of FBB on UVB-induced mitogen-activated protein kinase (MAPK) and nuclear factor kappa B (NF-κB) signaling in Hs68 cells. Cells were stimulated with UVB (100 mJ/cm^2^) after incubated with the indicated doses of LP, BB, BBL, and FBB for 24 h in Hs68 cells. (**A**) Western blot analysis of p-ERK1/2, p-JNK, p-p38, and p-NF-κB. (**B**) Each band was densitometrically quantified by image analysis. The band density was normalized to each total protein level followed by statistical analysis. *** *p* < 0.001 vs. N; ^###^ *p* < 0.001 vs. C. ^†^ *p* < 0.05 vs. BB. Values are means ± SEM of three independent experiments.

Based on the above data, we further investigated the molecular mechanism of the FBB effects. The MAPK and NF-κB signaling play an important role in UV-induced skin aging [5]. We investigated the inhibitory effect of FBB on the activities of these signaling cascades in Hs68 cells. FBB significantly inhibited UVB-induced phosphorylation of ERK1/2 (p44 and p42), JNK1/2 (p54 and p46), and p38 (Figure 3A, B). It is well established that a transcription factor, NF-κB, is activated upon UV irradiation [17]. It is also reported that UVB-induced skin photoaging can be attenuated by inhibiting NF-κB activation [18,19]. NF-κB was phosphorylated by UVB irradiation in Hs68 cells, which are significantly reduced by FBB (Figure 3A, B). LP and BBL also showed regulatory effects, similar to or lower than FBB, on UVB-induced MAPK and NF-κB activation. 

### 3.5. FBB Inhibits UVB-Induced Wrinkle Formation and COLs Degradation in SKH-1 Mice

**Figure 4:**
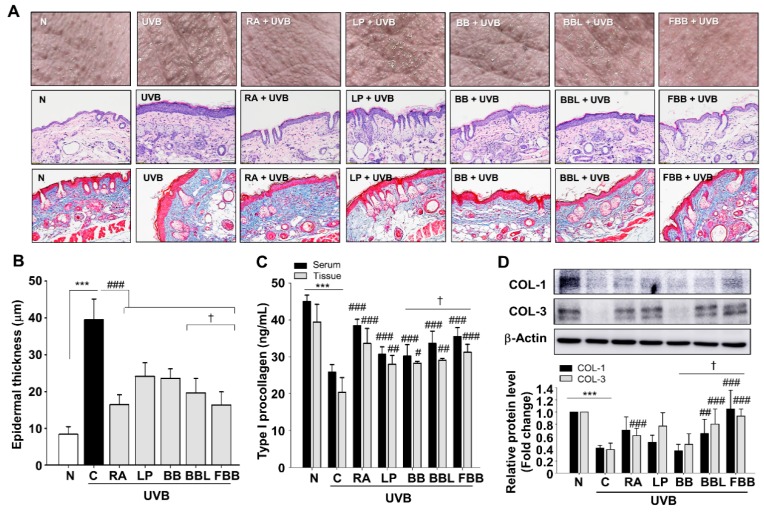
FBB protects UVB-induced wrinkle formation and collagen degradation in the dorsal skin of hairless mice. (**A**) Upper panel, representative dorsal skin surfaces from all groups of hairless mice skin after UVB-irradiation; Middle panel, hematoxylin and eosin staining of the dorsal skins; Lower panel, Masson’s trichrome staining for collagen fibers. Collagen fibers appear blue. (**B**) Epidermis thickness of different groups. The magnification was ×200. (**C**) Type I procollagen levels were measured in serum and tissue lysates from all groups using ELISA kit. (**D**) The expression level of COL-1 and -3 proteins were detected by western blotting. Each band was densitometrically quantified by image analysis. The band density was normalized to β-actin followed by statistical analysis. *** *p* < 0.001 vs. N; ^#^ *p* < 0.05, ^##^ *p* < 0.01, ^###^ *p* < 0.001 vs. C. ^†^ *p* < 0.05 vs. BB. Values are means ± SEM of three independent experiments.

UVB irradiation induces excessive epidermal proliferation and thickness that contribute to skin roughness [20]. To investigate the effect of FBB on wrinkle formation, the dorsal skins of hairless mice were exposed UVB with LP, BB, BBL, and FBB for 4 weeks. RA (0.01%) was used as a positive control. As shown in Figure 4A, UV exposure for 4 weeks triggered skin thickening and coarse wrinkles in the model mice. However, these macroscopic changes of UV irradiated skin were ameliorated in the FBB-administered groups (Figure 4A, upper panel). The skin tissue sections were subjected to H&E staining. Skin in UV irradiation group displayed typical features of photoaging, such as irregular thickening of epidermal layer and even the presence of inflammatory infiltrates in corium and subcutis. However, FBB administration ameliorated these skin structure damages induced by UV exposure (Figure 4A, middle panel, and Figure 4B). To determine whether the treatment of FBB to UVB-irradiated SKH-1 mice affects COLs degradation, the type I procollagen levels in serum and tissue lysates, histological changes, and the protein expressions of COL-1 and COL-3 were analyzed in mice skin tissue of each group. Dorsal skins were stained with Masson’s trichrome. UVB irradiation resulted in the loose and uneven distribution of collagen fibers. Increased bundles of collagen fibers were observed in the FBB-treated group (Figure 4A, lower panel). FBB significantly maintained type I pro-collagen levels in serum as well as in tissue lysates (Figure 4C). Moreover, FBB inhibited UVB-induced COL-1 and COL-3 protein downregulations (Figure 4D). FBB significantly reserved the COLs degradation compared with BB. FBB administration shows more effective than LP, BB, and BBL, suggesting that BB may be made more potent by fermentation.

### 3.6. FBB Prevents UVB-Induced MMPs Production in SKH-1 Mice 

**Figure 5:**
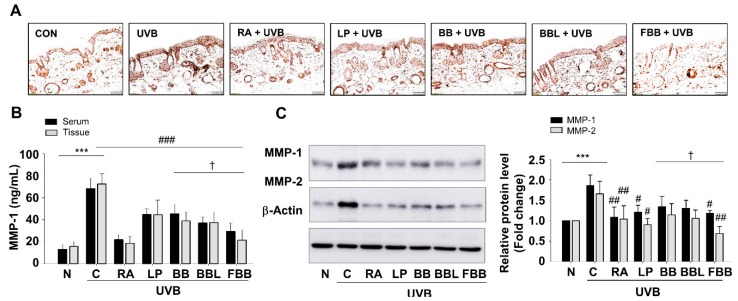
Suppression of MMP-1 expression in UVB-irradiated mouse skin by FBB. (**A**) Dorsal skin sections were immunostained with anti-MMP-1 antibody. The magnification was ×200. (**B**) MMP-1 levels were measured in serum and tissue lysates from all groups using ELISA kit. (**C**) The expression level of MMP-1 and -2 proteins were detected by western blotting. Each band was densitometrically quantified by image analysis. The band density was normalized to β-actin followed by statistical analysis. *** *p* < 0.001 vs. N; ^#^ *p* < 0.05, ^##^ *p* < 0.01, ^###^ *p* < 0.001 vs. C. ^†^ *p* < 0.05 vs. BB. Values are means ± SEM of three independent experiments.

To better understand whether the antiphotoaging effects of FBB, we tested the effect of FBB on MMPs increases in serum and tissue samples following UVB-irradiation. FBB treatment elicited a decrease in MMP-1 levels in serum as well as tissue lysates (Figure 5B). We observed the inhibitory effect of FBB on MMP-1 expression (Figure 5C), which was significantly increased in the epidermis, by immunostaining in the skin tissue of each group (Figure 5A). 

### 3.7. FBB Increases the Activity of Antioxidant Effcts

**Figure 6:**
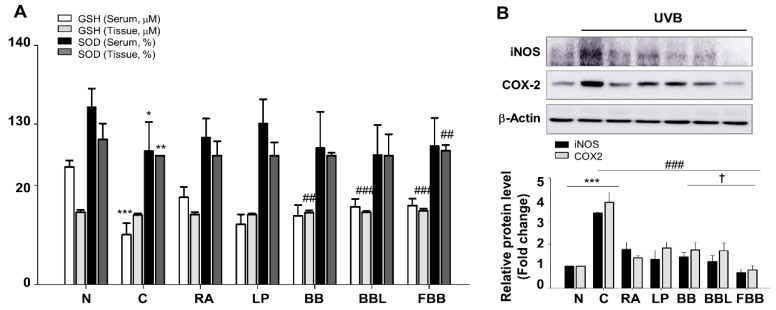
Effect of FBB on oxidative stress and iNOS and COX-2 protein expression in UVB-irradiated hairless mice. (**A**) Glutathione (GSH) concentrations and Superoxide dismutase (SOD) activities were used to measured antioxidant activities in serum and tissue lysates. (**B**) Immunoblot analysis of the proteins in skin homogenates of hairless mice was performed as Materials and Methods. The expression level of iNOS and COX-2 proteins. Each band was densitometrically quantified by image analysis. The band density was normalized to β-actin followed by statistical analysis. * *p* < 0.05, ** *p* < 0.01, *** *p* < 0.001 vs. N; ^##^ *p* < 0.01, ^###^ *p* < 0.001 vs. C. ^†^ *p* < 0.05 vs. BB. Values are means ± SEM of three independent experiments.

Oxidative stress could be involved in the photoaging process as a primary factor. Because the antioxidant activity of BB was increased after fermentation (Table 1), there is a possibility that administration of FBB promotes antioxidant capacity. To confirm this possibility, we assessed the antioxidant capacity in the serum and tissue on the basis of GSH and SOD activity. As shown in Figure 6A, GSH content significantly decreased following UV irradiation (*p* < 0.001) in serum, FBB treatment significantly increases the activity of GSH (*p* < 0.001) compared with the UVB group. In tissue lysates, GSH content was no significant difference between normal and UVB-irradiated group. Then we also assessed antioxidative capacity on the basis of the activity of SOD, FBB significantly increased the SOD activity in tissue lysate (*p* < 0.01). There was no significant difference between UVB and FBB administration groups in serum (Figure 6A). These data suggest that FBB shows anti-oxidative effects against UVB-induced oxidative stress in mice. The ROS by UV irradiation induces an inflammatory response, such as iNOS and COX-2, and damages the extracellular matrix. UVB irradiation showed a range of about 3.5–4.5-fold increase in iNOS and COX-2 expression in tissue lysate (Figure 6B). FBB treatment, however, had a significant effect on lowering iNOS and COX-2 levels (Figure 6B). The overall antioxidant capacity was higher in the FBB treatment group than in LP, BB, and BBL groups.

### 3.8. Effect of FBB on UVB-Mediated MAPK and NF-κB Signaling in SKH-1 Mice

**Figure 7:**
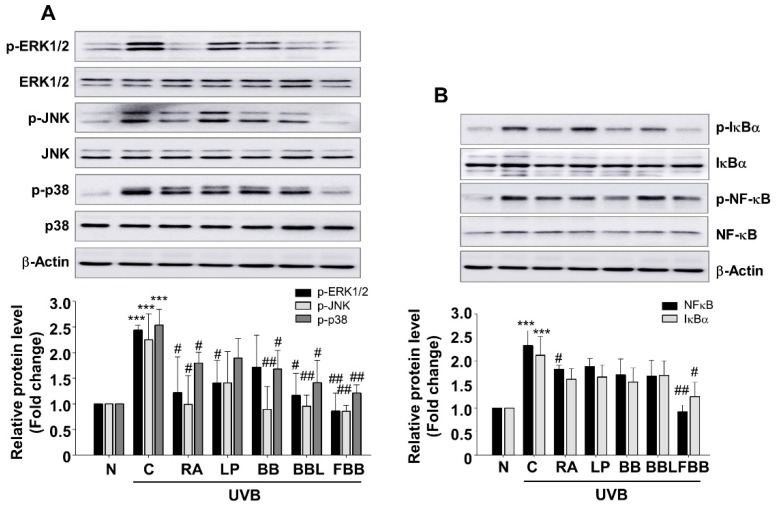
Effect of FBB on UVB-induced MAPKs and NF-κB signaling in hairless mice. (**A**) Western blot analysis of p-ERK1/2, p-JNK, and p-p38 expression in skin tissue lysates. (**B**) Western blot analysis of p-IκBα NF-κB and expression. Each band was densitometrically quantified by image analysis. The band density was normalized to each total protein followed by statistical analysis. *** *p* < 0.001 vs. N; ^#^ *p* < 0.05, ^##^ *p* < 0.01, ^###^ *p* < 0.001 vs. C. Values are means ± SEM of three independent experiments.

The pathway of MAPK signal transduction plays an important role in regulating immune response and inflammation, including matrix metalloproteinases (MMPs) expression [21]. UV-irradiation can activate and translocate NF-κB into the nucleus which is responsible for skin aging and collagen degradation. Our *in vitro* study showed that FBB inhibits UVB-induced phosphorylation of MAPKs and NF-κB in Hs68 cells (Figure 3). To determine whether UVB could induce activation of MAPKs and NF-κB under in vivo condition in SKH-1 hairless mice, western blot analysis was performed using skin tissue lysates. UVB exposure resulted in increased phosphorylation of ERK1/2, JNK1/2, and p38. Administration of FBB, prior to repeated UVB exposure, significantly inhibited the phosphorylation of ERK1/2, JNK1/2, and p38 (Figure 7A). As demonstrated by the relative density of the bands, treatment of FBB significantly inhibited UVB-induced phosphorylation of ERK1/2, JNK1/2, and p38, and no significant differences were observed between the BB and FBB groups. Activation of MAPKs causes the activations of several transcription factors, such as NF-κB. One of the critical events in NF-κB activation is its dissociation with subsequent degradation of inhibitory protein IκBα via phosphorylation. As shown in Figure 7B, FBB effectively regulated NF-κB and IκBα phosphorylation in UVB-induced mice skin. These results support that regulation of MAPK and NF-κB signaling by FBB may contribute to the protective effects on skin collagen destruction against UVB-irradiation in mice.

## 4. Discussion

The dermis of human skin contains a large number of fibroblasts, which are responsible for the generation of extracellular matrix (ECM). The functional properties of the skin depend on the integrity of collagen in the dermis. Loss of collagen matrix can be caused by decreased collagen synthesis or proteolysis by collagenase such as MMPs. UV irradiation accelerates skin aging, which is known to induce the expression of MMPs [3]. 

The beneficial properties of blackberry (*Rubus fruticosus* B.) to antioxidant, anti-inflammatory, and wound healing have been demonstrated because of their enriched polyphenols, flavonoids, and vitamins [22]. Studies showed that fermentation with probiotics promotes health benefits including allergic rhinitis, cardiovascular disease, type 2 diabetes, obesity, immune-related pathologies, and skin photoaging [7,23,24,25]. Myrtle berries homogenate, fermented with *L. plantarum* C2, was found to contain the increased antioxidant activity via increased concentrations of total phenols and flavonoids [25]. *Agastache rugosa* Kuntze (Korean mint), fermented with *L. rhamnosus* HK-9, showed higher attenuating activity on the UVB-induced ROS generation and photoaging in HaCaT keratinocytes [24]. Thus, in the present study, we investigated whether fermented BB with *L. plantarum* JBMI F5 can be used to regulate UVB induced photoaging in human foreskin fibroblast and hairless mice. 

We analyzed changes in biochemical and antioxidant effects after fermentation. As shown in Table 1, the TPC and TFC were significantly increased after fermentation with *L. plantarum* JBMI F5, which were increased to 18.96 ± 0.25 and 0.38 ± 0.02 mg/g, respectively. Additionally, vitamin C was improved approximately 6.5-fold in FBB compared to BB (Table 1). 

FBB treatment significantly inhibits UVB-induced collagenase activation and collagen destruction in human fibroblast Hs68 cells. Oral administration of FBB also suppressed UVB-induced collagen degradation and MMPs activation, thereby inhibiting wrinkle formation and epidermal thickening in the dorsal skin of hairless mice. In the present study, FBB was found to scavenge reactive oxygen radicals (Table 1) and significantly increase antioxidants, GSH and SOD, in UVB-induced hairless mouse serum and skin (Figure 6). ROS generation triggered by UV plays an important role in MAPK mediated signal conversion. In addition, ROS increases MMP-1 expression in human dermal fibroblasts, and ROS scavengers inhibit UV-induced MMP-1 expression. Recently, it was reported that UV rapidly and significantly increases H_2_O_2_ levels in human skin in vivo, suggesting that an early increase in ROS may participate in the triggering of the MAPK cascade and that topical treatment with antioxidants may interrupt the activation of MAPK pathways and thus inhibit UV-induced MMP expression in human skin in vivo. Families of MAPKs include p38, as well as ERK and JNK, all of which exhibit extensive crosstalk among themselves [5]. The accumulation of ROS due to the weakening catalase can be an important aspect of the MAPK signaling changes that lead to skin aging and photoaging in human skin in vivo [26]. 

NF-κB is a decisive factor for the immuno-inflammatory responses and has also been involved in various skin diseases including allergic dermatitis, psoriasis vulgaris, and skin cancer. Accumulated evidence shows that NF-κB is one of the most vulnerable targets activated by UVB [27,28]. NF-κB exists in the cytoplasm of the majority of cells as homo- or hetero-dimers of a family of structurally related proteins known as Rel or Rel/NF-κB. Cytoplasmic sequestration of NF-κB is regulated by its binding to an inhibitory protein known as inhibitor-kappa B (IκB). Signals that induce transcriptional activation of NF-κB dissociate to the nucleus by UV irradiation. UVB irradiation stimulates IκB phosphorylation and dissociates IκB and NF-κB. Additional steps in the sequence of the phosphorylation of the NF-κB signaling leads to NF-κB activation [5]. 

In our results, the phosphorylation of MAPK and NF-κB was increased by UVB irradiation in Hs68 cells and hairless mouse skin and FBB remarkably reduced these levels. Furthermore, FBB significantly reduced the expressions of the COX-2 and iNOS proteins in UVB-induced mouse skin. The transcription of several MMP family members is strongly regulated by NF-κB lead to collagen degradation. The MMP-1 (collagenase 1) and MMP-2 (gelatinase-A) are responsible for COL-1 and -2 cleavage [29]. MMP-1 is mainly responsible for degradation of COL-1, which represents structural support of the dermis, and is its primary component. MMP-1 is considered an effective biomarker of photoaging, and its upregulation accelerates skin aging, thus leading to wrinkle formation and impairment of skin integrity. It was reported that MMP-2 protein expression was increased by stress-induced ex vivo human skin model, leading to reduction of collagen content [30]. 

The present study revealed that FBB significantly normalized the reduction of type I procollagen content and MMP-1 increase in Hs68 and hairless mouse serum and skin. In addition, FBB inhibited UVB-mediated COL-1 and -3 protein degradation as well as MMP-1 and -2 protein expression in Hs68 and hairless mouse skin. In addition, FBB inhibited protein expression of iNOS, COX-2, and phosphorylation of MAPK and NF-κB induced by UVB in Hs68 and hairless mouse. FBB may be used in skincare in the future. An important finding in the present study is that the blackberries fermented by *L. plantarum* JBMI F5 have an improved effect compared to the original blackberry. The results showed that the fermented blackberry had a better effect than the blackberry original, *L. plantarum* JBMI F5, as well as LP inoculated BB. In conclusion, FBB is a potential therapeutic agent for the prevention and treatment for photoaging of the skin.

## Figures and Tables

**Table 1:** Biochemical contents and antioxidant effects of samples.

	TPC (mg/g)	TFC (mg/g)	VC (mg/100 g)	DPPH Radical Scavenging Activity (%)	ABTS Radical Scavenging Activity (%)
BB	18.96 ± 0.25	0.24 ± 0.09	51.05 ± 3.36	5.17 ± 0.98	6.06 ± 0.45
FBB	28.86 ± 0.25 ***	0.38 ± 0.02 *	282.56 ± 34.63 ***	14.19 ± 0.32 ***	33.06 ± 0.88 ***
AA	NA	NA	NA	94.24 ± 0.13	NA
Trolox	NA	NA	NA	NA	94.62 ± 0.08

Samples were assayed at 5 mg/mL final concentration for antioxidant effects; and ascorbic acid and trolox was assayed at 0.1 mg/mL final concentration. Values are means ± SD of three independent experiments. * *p* < 0.005; *** *p* < 0.001 vs. BB. TPC; total polyphenol content; TFC; total flavonoid content; BB; Black berry; FBB; fermented BB; VC; vitamin C content; ABTS; 2,2′-azino-bis(3-ethylbenzothiazoline-6-sulphonic acid); DPPH; 2,2-diphenyl-1-pycrylhydrazyl; AA; Ascorbic acid; NA; Not Applicable.
